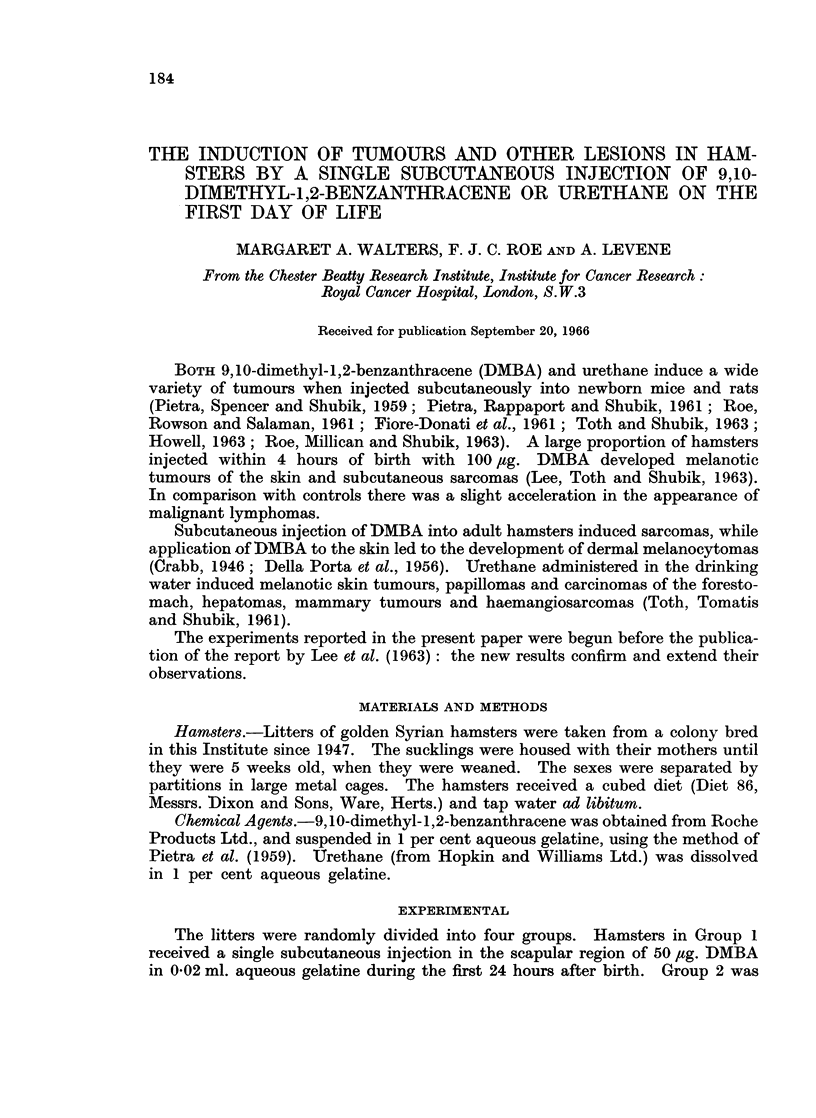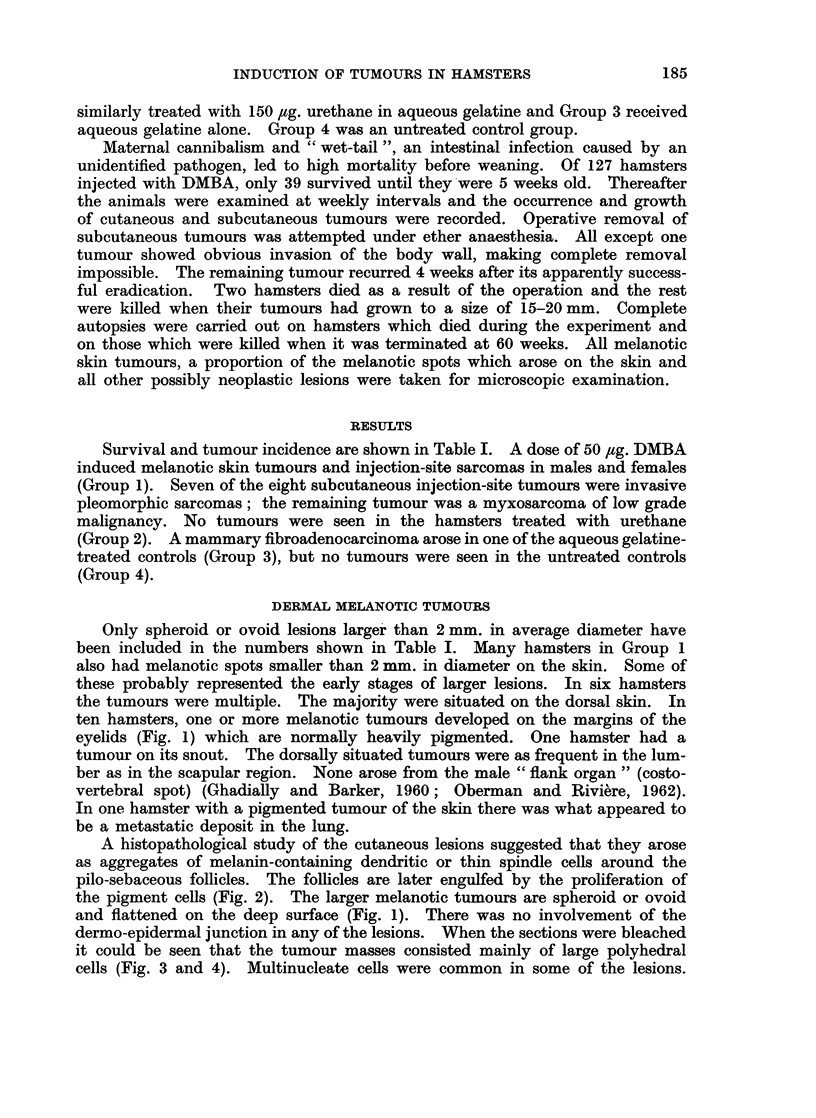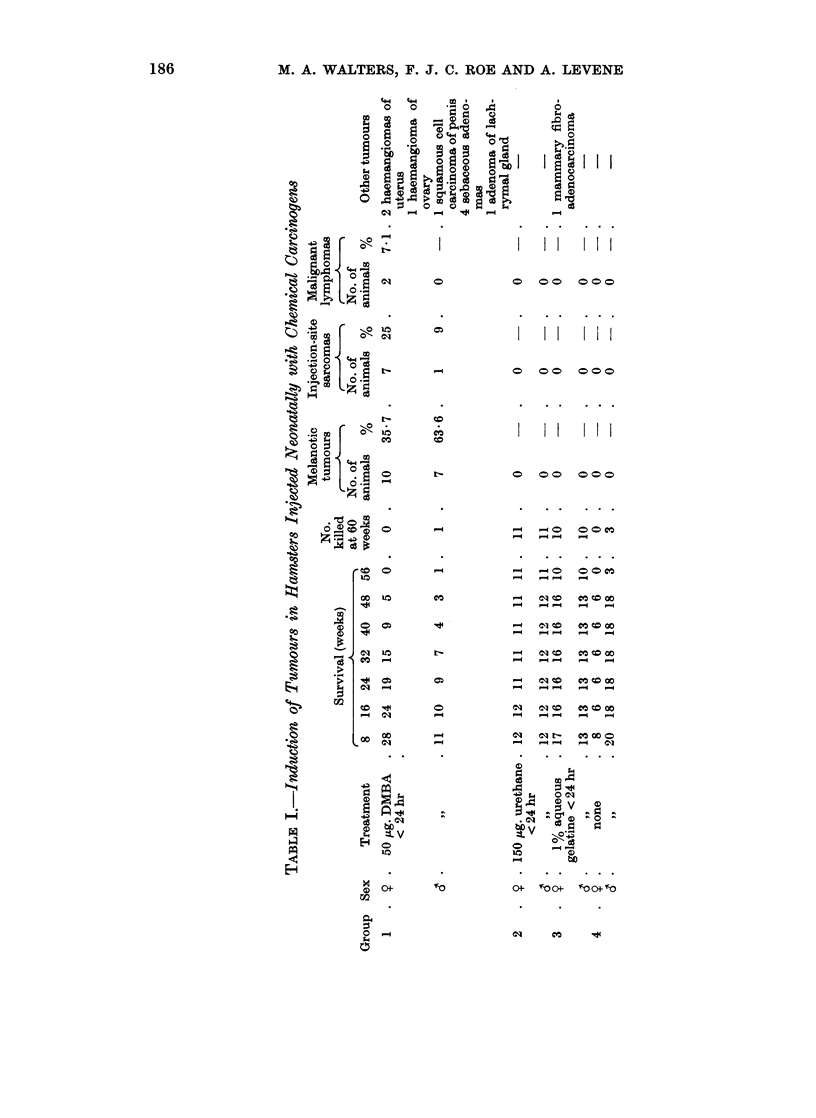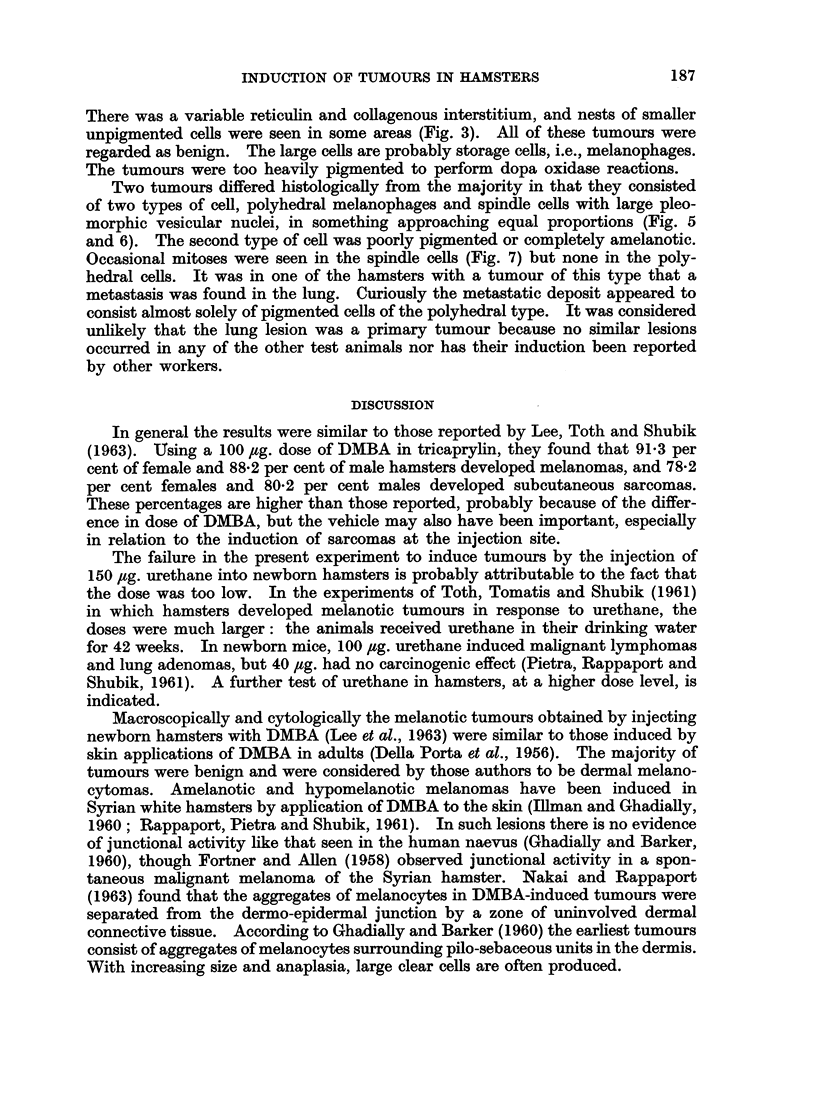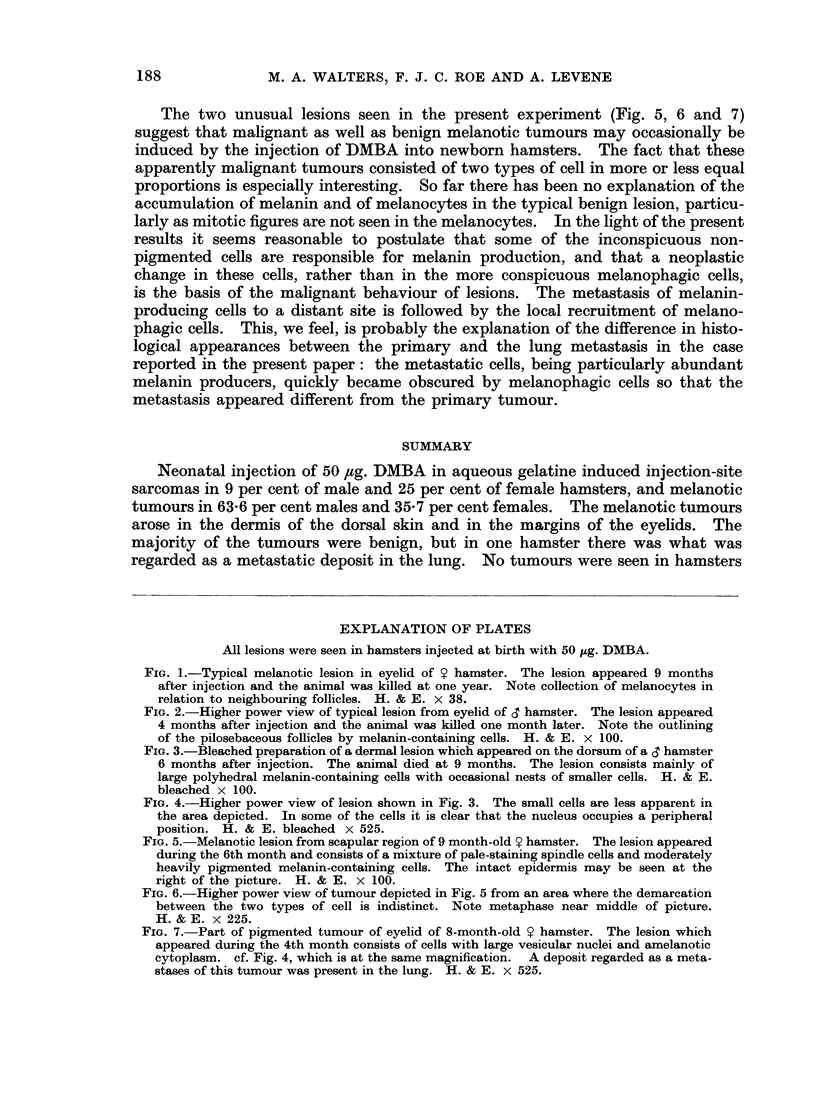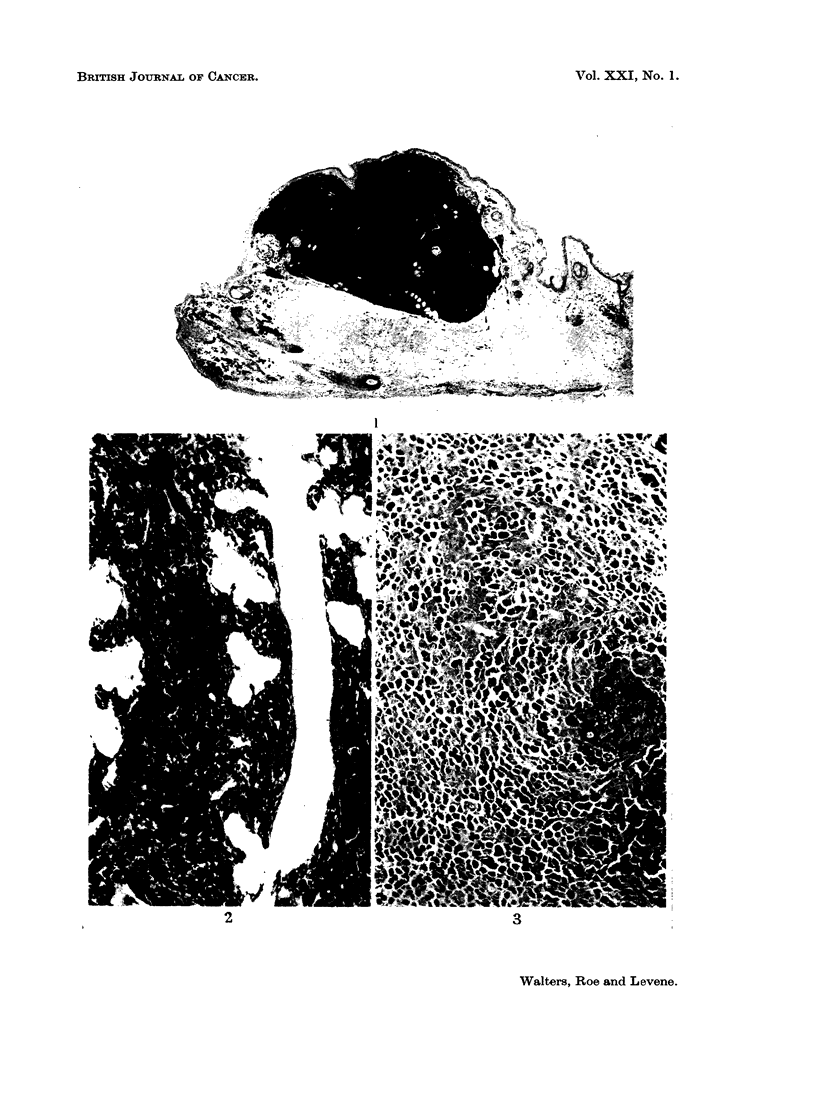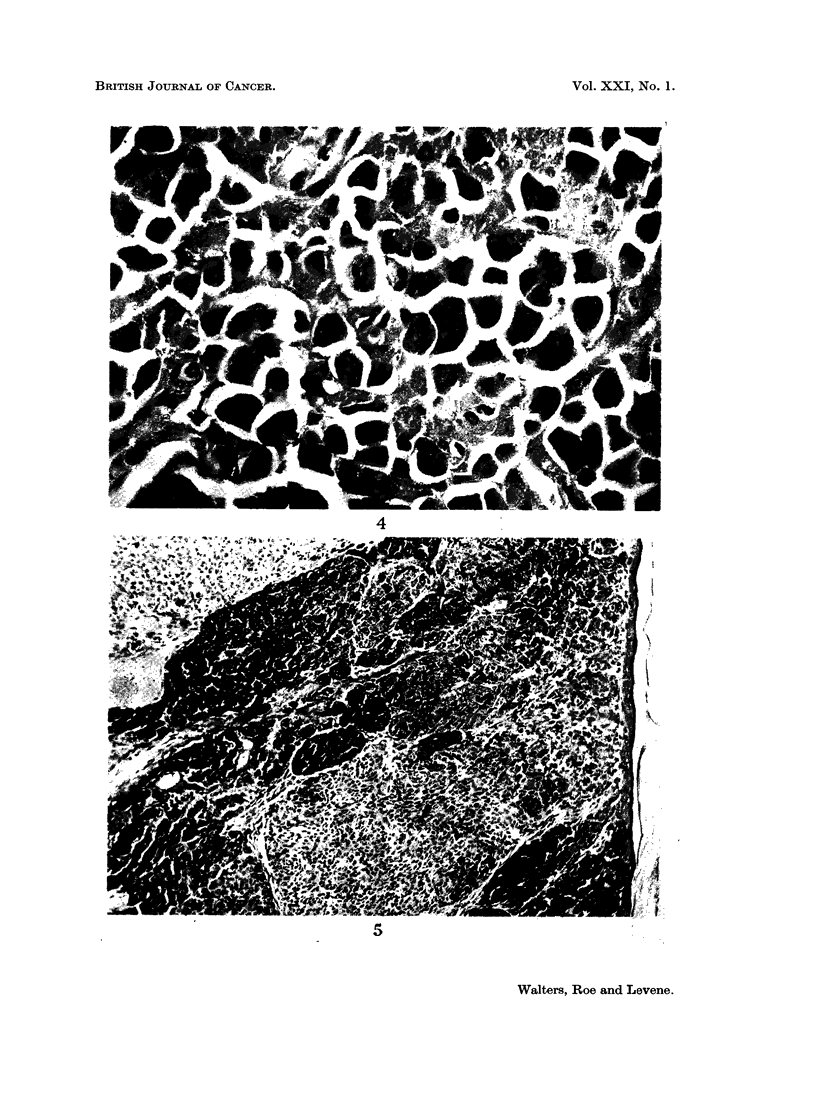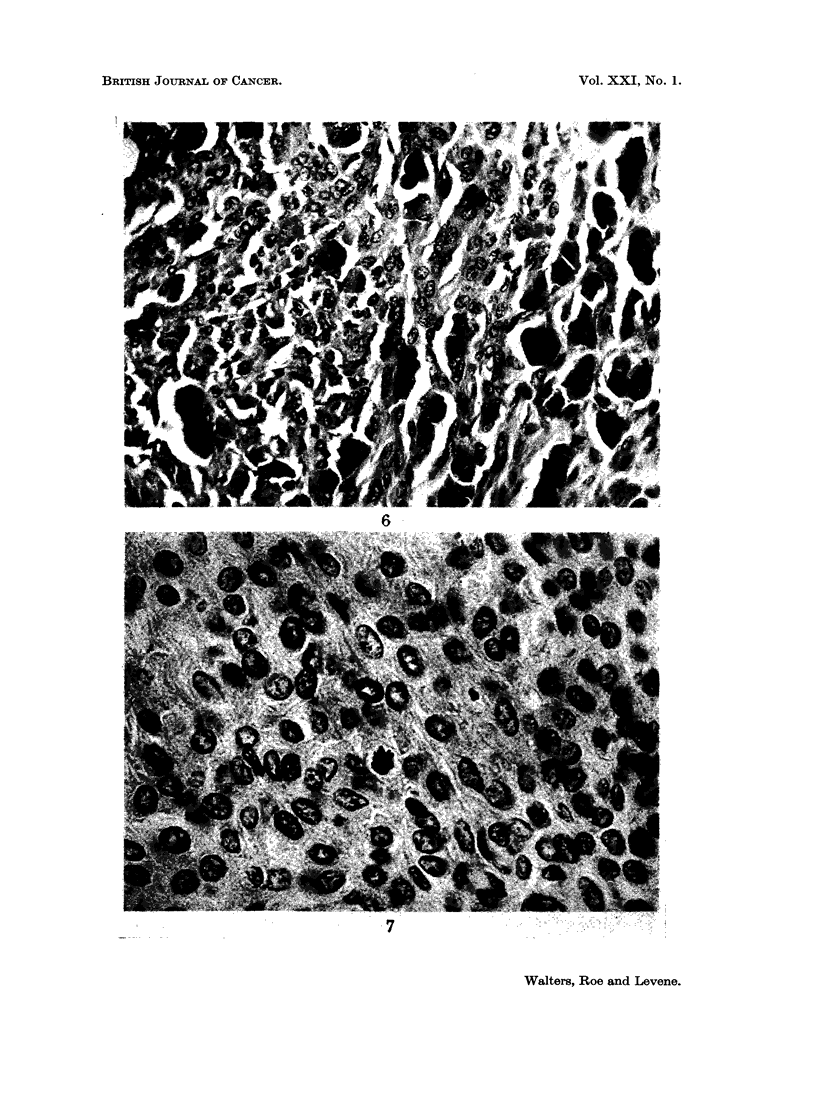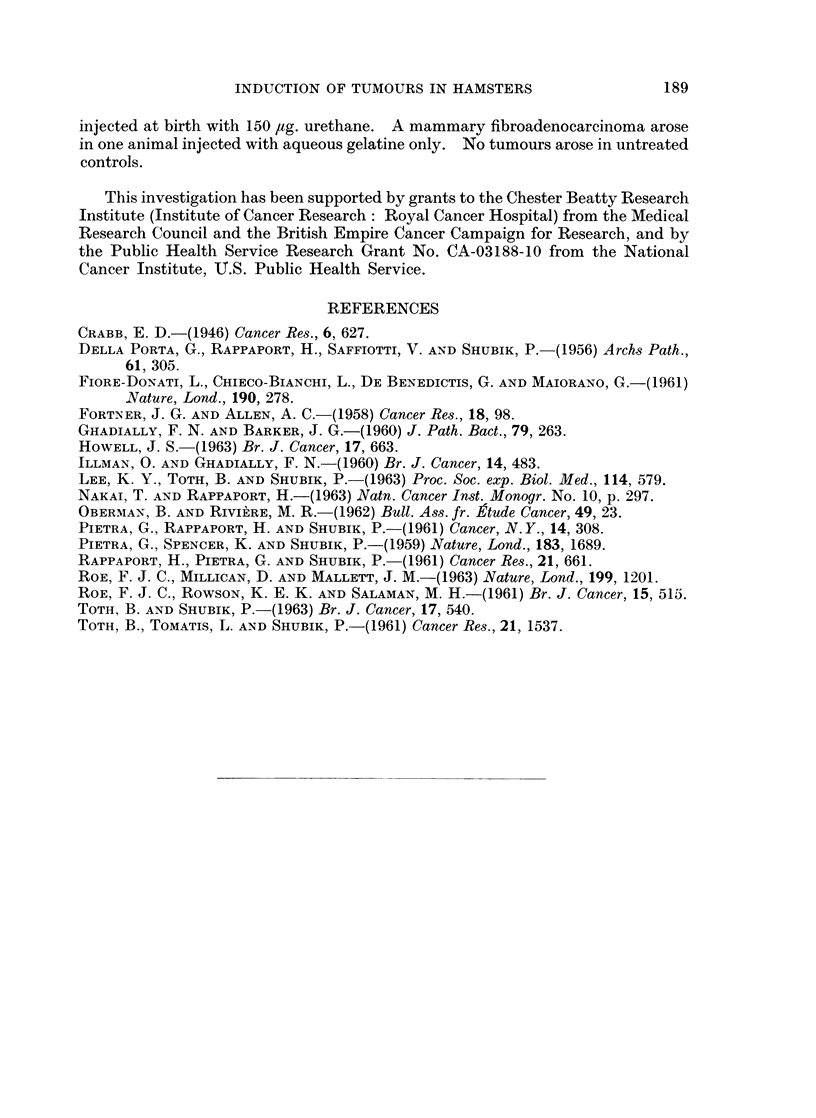# The induction of tumours and other lesions in hamsters by a single subcutaneous injection of 9,10-dimethyl-1,2-benzanthracene or urethane on the first day of life.

**DOI:** 10.1038/bjc.1967.19

**Published:** 1967-03

**Authors:** M. A. Walters, F. J. Roe, A. Levene

## Abstract

**Images:**


### THE INDUCTION OF TUMOURS AND OTHER LESIONS IN HAMSTERS BY A SINGLE SUBCUTANEOUS INJECTION OF 9,10-DIMETHYL-1,2-BENZANTHRACENE OR URETHANE ON THE FIRST DAY OF LIFE

MARGARET A. WALTERS, F. J. C. ROE AND A. LEVENE

*From the Chester Beatty Research Institute, Institute for Cancer Research: Royal Cancer Hospital, London, S.W.3*

Received for publication September 20, 1966

Both 9,10-dimethyl-1,2-benzanthracene (DMBA) and urethane induce a wide variety of tumours when injected subcutaneously into newborn mice and rats (Pietra, Spencer and Shubik, 1959; Pietra, Rappaport and Shubik, 1961; Roe, Rowson and Salaman, 1961; Fiore-Donati *et al.*, 1961; Toth and Shubik, 1963; Howell, 1963; Roe, Millican and Shubik, 1963). A large proportion of hamsters injected within 4 hours of birth with 100 $\mu$g. DMBA developed melanotic tumours of the skin and subcutaneous sarcomas (Lee, Toth and Shubik, 1963). In comparison with controls there was a slight acceleration in the appearance of malignant lymphomas.

Subcutaneous injection of DMBA into adult hamsters induced sarcomas, while application of DMBA to the skin led to the development of dermal melanocytomas (Crabb, 1946; Della Porta *et al.*, 1956). Urethane administered in the drinking water induced melanotic skin tumours, papillomas and carcinomas of the forestomach, hepatomas, mammary tumours and haemangiosarcomas (Toth, Tomatis and Shubik, 1961).

The experiments reported in the present paper were begun before the publication of the report by Lee *et al.* (1963): the new results confirm and extend their observations.

### MATERIALS AND METHODS

*Hamsters.*—Litters of golden Syrian hamsters were taken from a colony bred in this Institute since 1947. The sucklings were housed with their mothers until they were 5 weeks old, when they were weaned. The sexes were separated by partitions in large metal cages. The hamsters received a cubed diet (Diet 86, Messrs. Dixon and Sons, Ware, Herts.) and tap water *ad libitum*.

*Chemical Agents.*—9,10-dimethyl-1,2-benzanthracene was obtained from Roche Products Ltd., and suspended in 1 per cent aqueous gelatine, using the method of Pietra *et al.* (1959). Urethane (from Hopkin and Williams Ltd.) was dissolved in 1 per cent aqueous gelatine.

### EXPERIMENTAL

The litters were randomly divided into four groups. Hamsters in Group 1 received a single subcutaneous injection in the scapular region of 50 $\mu$g. DMBA in 0·02 ml. aqueous gelatine during the first 24 hours after birth. Group 2 was similarly treated with 150 $\mu$g. urethane in aqueous gelatine and Group 3 received aqueous gelatine alone. Group 4 was an untreated control group.

Maternal cannibalism and " wet-tail ", an intestinal infection caused by an unidentified pathogen, led to high mortality before weaning. Of 127 hamsters injected with DMBA, only 39 survived until they were 5 weeks old. Thereafter the animals were examined at weekly intervals and the occurrence and growth of cutaneous and subcutaneous tumours were recorded. Operative removal of subcutaneous tumours was attempted under ether anaesthesia. All except one tumour showed obvious invasion of the body wall, making complete removal impossible. The remaining tumour recurred 4 weeks after its apparently successful eradication. Two hamsters died as a result of the operation and the rest were killed when their tumours had grown to a size of 15–20 mm. Complete autopsies were carried out on hamsters which died during the experiment and on those which were killed when it was terminated at 60 weeks. All melanotic skin tumours, a proportion of the melanotic spots which arose on the skin and all other possibly neoplastic lesions were taken for microscopic examination.

### RESULTS

Survival and tumour incidence are shown in Table I. A dose of 50 $\mu$g. DMBA induced melanotic skin tumours and injection-site sarcomas in males and females (Group 1). Seven of the eight subcutaneous injection-site tumours were invasive pleomorphic sarcomas ; the remaining tumour was a myxosarcoma of low grade malignancy. No tumours were seen in the hamsters treated with urethane (Group 2). A mammary fibroadenocarcinoma arose in one of the aqueous gelatine-treated controls (Group 3), but no tumours were seen in the untreated controls (Group 4).

### DERMAL MELANOTIC TUMOURS

Only spheroid or ovoid lesions larger than 2 mm. in average diameter have been included in the numbers shown in Table I. Many hamsters in Group 1 also had melanotic spots smaller than 2 mm. in diameter on the skin. Some of these probably represented the early stages of larger lesions. In six hamsters the tumours were multiple. The majority were situated on the dorsal skin. In ten hamsters, one or more melanotic tumours developed on the margins of the eyelids (Fig. 1) which are normally heavily pigmented. One hamster had a tumour on its snout. The dorsally situated tumours were as frequent in the lumber as in the scapular region. None arose from the male " flank organ " (costo-vertebral spot) (Ghadially and Barker, 1960 ; Oberman and Rivière, 1962). In one hamster with a pigmented tumour of the skin there was what appeared to be a metastatic deposit in the lung.

A histopathological study of the cutaneous lesions suggested that they arose as aggregates of melanin-containing dendritic or thin spindle cells around the pilo-sebaceous follicles. The follicles are later engulfed by the proliferation of the pigment cells (Fig. 2). The larger melanotic tumours are spheroid or ovoid and flattened on the deep surface (Fig. 1). There was no involvement of the dermo-epidermal junction in any of the lesions. When the sections were bleached it could be seen that the tumour masses consisted mainly of large polyhedral cells (Fig. 3 and 4). Multinucleate cells were common in some of the lesions.

TABLE I.—*Induction of Tumours in Hamsters Injected Neonatally with Chemical Carcinogens*

| Group | Sex | Treatment | Survival (weeks) 8 | 16 | 24 | 32 | 40 | 48 | 56 | No. killed at 60 weeks | Melanotic tumours No. of animals | % | Injection-site sarcomas No. of animals | % | Malignant lymphomas No. of animals | % | Other tumours |
|---|---|---|---|---|---|---|---|---|---|---|---|---|---|---|---|---|---|
| 1 | ♀ | 50 μg. DMBA < 24 hr | 28 | 24 | 19 | 15 | 9 | 5 | 0 | 0 | 10 | 35·7 | 7 | 25 | 2 | 7·1 | 2 haemangiomas of uterus<br>1 haemangioma of ovary |
| | ♂ | ,, | 11 | 10 | 9 | 7 | 4 | 3 | 1 | 1 | 7 | 63·6 | 1 | 9 | 0 | — | 1 squamous cell carcinoma of penis<br>4 sebaceous adenomas<br>1 adenoma of lachrymal gland |
| 2 | ♀ | 150 μg. urethane < 24 hr | 12 | 12 | 11 | 11 | 11 | 11 | 11 | 11 | 0 | — | 0 | — | 0 | — | — |
| | ♂ | ,, | 12 | 12 | 12 | 12 | 12 | 12 | 11 | 11 | 0 | — | 0 | — | 0 | — | — |
| 3 | ♀ | 1% aqueous gelatine < 24 hr | 17 | 16 | 16 | 16 | 16 | 16 | 10 | 10 | 0 | — | 0 | — | 0 | — | 1 mammary fibroadenocarcinoma |
| | ♂ | ,, | 13 | 13 | 13 | 13 | 13 | 13 | 10 | 10 | 0 | — | 0 | — | 0 | — | — |
| 4 | ♀ | none | 8 | 6 | 6 | 6 | 6 | 6 | 0 | 0 | 0 | — | 0 | — | 0 | — | — |
| | ♂ | ,, | 20 | 18 | 18 | 18 | 18 | 18 | 3 | 3 | 0 | — | 0 | — | 0 | — | — |

There was a variable reticulin and collagenous interstitium, and nests of smaller unpigmented cells were seen in some areas (Fig. 3). All of these tumours were regarded as benign. The large cells are probably storage cells, i.e., melanophages. The tumours were too heavily pigmented to perform dopa oxidase reactions.

Two tumours differed histologically from the majority in that they consisted of two types of cell, polyhedral melanophages and spindle cells with large pleomorphic vesicular nuclei, in something approaching equal proportions (Fig. 5 and 6). The second type of cell was poorly pigmented or completely amelanotic. Occasional mitoses were seen in the spindle cells (Fig. 7) but none in the polyhedral cells. It was in one of the hamsters with a tumour of this type that a metastasis was found in the lung. Curiously the metastatic deposit appeared to consist almost solely of pigmented cells of the polyhedral type. It was considered unlikely that the lung lesion was a primary tumour because no similar lesions occurred in any of the other test animals nor has their induction been reported by other workers.

### DISCUSSION

In general the results were similar to those reported by Lee, Toth and Shubik (1963). Using a 100 $\mu$g. dose of DMBA in tricaprylin, they found that 91·3 per cent of female and 88·2 per cent of male hamsters developed melanomas, and 78·2 per cent females and 80·2 per cent males developed subcutaneous sarcomas. These percentages are higher than those reported, probably because of the difference in dose of DMBA, but the vehicle may also have been important, especially in relation to the induction of sarcomas at the injection site.

The failure in the present experiment to induce tumours by the injection of 150 $\mu$g. urethane into newborn hamsters is probably attributable to the fact that the dose was too low. In the experiments of Toth, Tomatis and Shubik (1961) in which hamsters developed melanotic tumours in response to urethane, the doses were much larger : the animals received urethane in their drinking water for 42 weeks. In newborn mice, 100 $\mu$g. urethane induced malignant lymphomas and lung adenomas, but 40 $\mu$g. had no carcinogenic effect (Pietra, Rappaport and Shubik, 1961). A further test of urethane in hamsters, at a higher dose level, is indicated.

Macroscopically and cytologically the melanotic tumours obtained by injecting newborn hamsters with DMBA (Lee *et al.*, 1963) were similar to those induced by skin applications of DMBA in adults (Della Porta *et al.*, 1956). The majority of tumours were benign and were considered by those authors to be dermal melanocytomas. Amelanotic and hypomelanotic melanomas have been induced in Syrian white hamsters by application of DMBA to the skin (Illman and Ghadially, 1960 ; Rappaport, Pietra and Shubik, 1961). In such lesions there is no evidence of junctional activity like that seen in the human naevus (Ghadially and Barker, 1960), though Fortner and Allen (1958) observed junctional activity in a spontaneous malignant melanoma of the Syrian hamster. Nakai and Rappaport (1963) found that the aggregates of melanocytes in DMBA-induced tumours were separated from the dermo-epidermal junction by a zone of uninvolved dermal connective tissue. According to Ghadially and Barker (1960) the earliest tumours consist of aggregates of melanocytes surrounding pilo-sebaceous units in the dermis. With increasing size and anaplasia, large clear cells are often produced.

The two unusual lesions seen in the present experiment (Fig. 5, 6 and 7) suggest that malignant as well as benign melanotic tumours may occasionally be induced by the injection of DMBA into newborn hamsters. The fact that these apparently malignant tumours consisted of two types of cell in more or less equal proportions is especially interesting. So far there has been no explanation of the accumulation of melanin and of melanocytes in the typical benign lesion, particularly as mitotic figures are not seen in the melanocytes. In the light of the present results it seems reasonable to postulate that some of the inconspicuous non-pigmented cells are responsible for melanin production, and that a neoplastic change in these cells, rather than in the more conspicuous melanophagic cells, is the basis of the malignant behaviour of lesions. The metastasis of melanin-producing cells to a distant site is followed by the local recruitment of melanophagic cells. This, we feel, is probably the explanation of the difference in histological appearances between the primary and the lung metastasis in the case reported in the present paper : the metastatic cells, being particularly abundant melanin producers, quickly became obscured by melanophagic cells so that the metastasis appeared different from the primary tumour.

### SUMMARY

Neonatal injection of 50 $\mu$g. DMBA in aqueous gelatine induced injection-site sarcomas in 9 per cent of male and 25 per cent of female hamsters, and melanotic tumours in 63·6 per cent males and 35·7 per cent females. The melanotic tumours arose in the dermis of the dorsal skin and in the margins of the eyelids. The majority of the tumours were benign, but in one hamster there was what was regarded as a metastatic deposit in the lung. No tumours were seen in hamsters

---

### EXPLANATION OF PLATES

All lesions were seen in hamsters injected at birth with 50 $\mu$g. DMBA.

FIG. 1.—Typical melanotic lesion in eyelid of ♀ hamster. The lesion appeared 9 months after injection and the animal was killed at one year. Note collection of melanocytes in relation to neighbouring follicles. H. & E. × 38.

FIG. 2.—Higher power view of typical lesion from eyelid of ♂ hamster. The lesion appeared 4 months after injection and the animal was killed one month later. Note the outlining of the pilosebaceous follicles by melanin-containing cells. H. & E. × 100.

FIG. 3.—Bleached preparation of a dermal lesion which appeared on the dorsum of a ♂ hamster 6 months after injection. The animal died at 9 months. The lesion consists mainly of large polyhedral melanin-containing cells with occasional nests of smaller cells. H. & E. bleached × 100.

FIG. 4.—Higher power view of lesion shown in Fig. 3. The small cells are less apparent in the area depicted. In some of the cells it is clear that the nucleus occupies a peripheral position. H. & E. bleached × 525.

FIG. 5.—Melanotic lesion from scapular region of 9 month-old ♀ hamster. The lesion appeared during the 6th month and consists of a mixture of pale-staining spindle cells and moderately heavily pigmented melanin-containing cells. The intact epidermis may be seen at the right of the picture. H. & E. × 100.

FIG. 6.—Higher power view of tumour depicted in Fig. 5 from an area where the demarcation between the two types of cell is indistinct. Note metaphase near middle of picture. H. & E. × 225.

FIG. 7.—Part of pigmented tumour of eyelid of 8-month-old ♀ hamster. The lesion which appeared during the 4th month consists of cells with large vesicular nuclei and amelanotic cytoplasm. cf. Fig. 4, which is at the same magnification. A deposit regarded as a metastases of this tumour was present in the lung. H. & E. × 525.

1

2

3

Walters, Roe and Levene.

4

5

Walters, Roe and Levene.

6

7

Walters, Roe and Levene.

injected at birth with 150 $\mu$g. urethane. A mammary fibroadenocarcinoma arose in one animal injected with aqueous gelatine only. No tumours arose in untreated controls.

This investigation has been supported by grants to the Chester Beatty Research Institute (Institute of Cancer Research : Royal Cancer Hospital) from the Medical Research Council and the British Empire Cancer Campaign for Research, and by the Public Health Service Research Grant No. CA-03188-10 from the National Cancer Institute, U.S. Public Health Service.

### REFERENCES

CRABB, E. D.—(1946) *Cancer Res.*, **6**, 627.
DELLA PORTA, G., RAPPAPORT, H., SAFFIOTTI, V. AND SHUBIK, P.—(1956) *Archs Path.*, **61**, 305.
FIORE-DONATI, L., CHIECO-BIANCHI, L., DE BENEDICTIS, G. AND MAIORANO, G.—(1961) *Nature, Lond.*, **190**, 278.
FORTNER, J. G. AND ALLEN, A. C.—(1958) *Cancer Res.*, **18**, 98.
GHADIALLY, F. N. AND BARKER, J. G.—(1960) *J. Path. Bact.*, **79**, 263.
HOWELL, J. S.—(1963) *Br. J. Cancer*, **17**, 663.
ILLMAN, O. AND GHADIALLY, F. N.—(1960) *Br. J. Cancer*, **14**, 483.
LEE, K. Y., TOTH, B. AND SHUBIK, P.—(1963) *Proc. Soc. exp. Biol. Med.*, **114**, 579.
NAKAI, T. AND RAPPAPORT, H.—(1963) *Natn. Cancer Inst. Monogr.* No. 10, p. 297.
OBERMAN, B. AND RIVIÈRE, M. R.—(1962) *Bull. Ass. fr. Étude Cancer*, **49**, 23.
PIETRA, G., RAPPAPORT, H. AND SHUBIK, P.—(1961) *Cancer, N.Y.*, **14**, 308.
PIETRA, G., SPENCER, K. AND SHUBIK, P.—(1959) *Nature, Lond.*, **183**, 1689.
RAPPAPORT, H., PIETRA, G. AND SHUBIK, P.—(1961) *Cancer Res.*, **21**, 661.
ROE, F. J. C., MILLICAN, D. AND MALLETT, J. M.—(1963) *Nature, Lond.*, **199**, 1201.
ROE, F. J. C., ROWSON, K. E. K. AND SALAMAN, M. H.—(1961) *Br. J. Cancer*, **15**, 515.
TOTH, B. AND SHUBIK, P.—(1963) *Br. J. Cancer*, **17**, 540.
TOTH, B., TOMATIS, L. AND SHUBIK, P.—(1961) *Cancer Res.*, **21**, 1537.